# The Varying Price of Drugs

**Published:** 1907-02-09

**Authors:** 


### THE VARYING PRICE OF DRUGS.

There is perhaps no class of article in daily demand the value of which fluctuates so much as does the value of drugs. Bread, clothing, butchers' meat, and other necessaries of life maintain as a general rule a steady price, but the cost of drugs is constantly varying. Medical practitioners and chemists are sometimes at a loss to understand why one week a certain drug is quoted at such and such a price, and the following week at quite a different figure. Thus a few days ago the value of sulphonal was 3s. 6d. per lb; to-day it is quoted 11s. 6d. Less than three months back quinine sulphate could have been bought for 8d. per oz., while to-day it cannot be obtained much below 1s., even in large wholesale quantities. At one time santonin cost 5s. per lb.; now it costs 38s. 6d. Camphor is worth four times as much as formerly. Cascara sagrada has at one time and another been more than double and almost less than half its present price; and so one could continue to name drug after drug which has varied in value. It is only reasonable to inquire why these articles are continually varying in price, and the answers to such inquiries will be found not altogether uninteresting.

It should be stated at the outset that the cause of these fluctuations is, in every case, beyond the control of the wholesale druggist. He has just as little to do with the changes as have the medical practitioner, the chemist, and the general public, and in fact he is more seriously handicapped by varying prices than any of these. For instance, the wholesale druggist contracts to supply a hospital with certain drugs for a definite period; when he sends in his tender one or more of the drugs quoted may be half the price to which it may rise before the contract is terminated, and unless the contractor has had sufficient foresight to cover himself on making the contract he may be a heavy loser. It is obvious that the contractor will gain if prices go down, but the tendency of late has been in the other direction.

To a great extent the value of drugs like most other commodities is governed by the natural law of supply and demand; but not always. To find the cause of the "ups" and "downs" of the drug market one must examine each case on its own merits. Thus it would seem extremely unlikely that there has been such a divergence between the supply of and demand for sulphonal as to justify a sudden rise from 3s. 6d. to 11s. 6d. per lb.; in fact, there has been no such divergence, and the advance is due to a combination of makers, who, knowing the difficulties surrounding the manufacture of this product, feel assured that there is no need to fear competition. During the past few weeks the demand for quinine has increased enormously, owing to the prevalence of influenza; on the other hand, the supplies have been below the average, so that there is nothing to be surprised at in the increased value. The time when quinine cost 20s. an oz. will be in the memory of more than one who will find it difficult to understand to what the very substantial drop in price is due. It is common knowledge that at one time the world was practically dependent for its supply of cinchona bark on the trees which grew wild in the forests of Peru; not only was this bark poor in alkaloids, but the reckless system of collecting it resulted in the disappearance of the tree from many districts. The possibility of the extermination of the source of this valuable drug led the Dutch to attempt the cultivation of the tree, and the result of the attempt is that to-day the great bulk of our supply of cinchona bark is drawn from the island of Java. By cross-fertilisation the Dutch succeeded in creating a hybrid which yields four or five times as much quinine as the Peruvian variety. An idea of the importance of the cinchona trade of Java can be obtained from the fact that last year more than six and a half million kilogrammes of bark were shipped to Europe, representing something like 392 tons of quinine sulphate. The amount exported from Java does not represent the total production, for large quantities of bark are retained for the manufacture of quinine locally.

India, which is a very large consumer, grows its own bark and makes its own quinine. The reason then for the fall in price of quinine from 20s. to 1s. an oz. is the increase in supply of raw material richer in the alkaloid, the increase being altogether out of proportion to the increase in consumption, however great.

The fluctuation in value which is sometimes of daily occurrence may be due to several causes. In the ordinary course the value of quinine depends on the amount of the shipments from Java, which is declared at half-monthly intervals, and the price paid for bark in the public sales held in Amsterdam every fifth Thursday throughout the year. But there is another factor to be reckoned with—namely, speculation. Quinine is a great favourite with speculators, and when we hear that so many hundred thousand ounces have changed hands, it does not necessarily follow that that amount has passed into consumption. The buyer may never see his purchase, which before the end of the day may have passed to another at a price a fraction of a penny higher or lower. In this way the purchase may be bandied about from one to another.

Speculation may drive a coach-and-four through the law of supply and demand. So it often happens that values fluctuate even when production and consumption continue in the same ratio. Speculation is by no means restricted to the quinine market, but is a very important element in the market for many other drugs, the favourites of late having been cascara sagrada, cloves, and menthol.

By far the greatest proportion of our supply of camphor comes from Formosa, and this drug is a monopoly of the Japanese Government. Camphor has never been dearer than it is at present, owing to scarcity, which in turn is due to the fact that the camphor forests are overrun by savages. The probabilities are that, as these savages are gradually brought into a state of submission, the supply of camphor will increase. But the demand is also increasing, and it is possible that until synthetic camphor is produced in a sufficiently pure state and on a commercial scale, the Japanese product will not be obtainable at normal prices.

Santonin is manufactured by a syndicate in Russian Turkestan, which has a monopoly of this drug. When competition existed among makers the price was 5s.; now that santonin is in the hands of a monopoly it is worth nearly 40s. per lb. It often happens that a drug which is cheap one season is dear the next, and then again cheap a year or so later. Take, for instance, ipecacuanha, which is very dear at the present time. At the price it was selling two years or so ago the exporters probably found that there was not sufficient profit left for them after paying the collectors in Matogrosso, and packing and carting it to the coast. The result was that less was collected. Tempted by the high prices recently paid in London, larger supplies will be shipped in course of time, and values will recede. Then history will repeat itself.

Other drugs beside camphor and santonin are in the hands of monopolies, and a large number, including hypophosphites, caffeine, bromides, iodides, morphine, codeine, cocaine, mercurials, salicylates, chloral hydrate, phenazone, and many other drugs are managed by combinations or conventions. It sometimes happens that when a maker outside a convention comes into the market as a competitor, a period of low prices is experienced, the period lasting until such time as the competitor ceases to exist, or joins the combination.

It will be gathered from these few notes that the chief causes of the varying value of drugs are: under or over supply, increased or decreased demand, monopolies, speculation, and combination.

